# Urban cultivation in allotments maintains soil qualities adversely affected by conventional agriculture

**DOI:** 10.1111/1365-2664.12254

**Published:** 2014-04-24

**Authors:** Jill L. Edmondson, Zoe G. Davies, Kevin J. Gaston, Jonathan R. Leake

**Affiliations:** ^1^ Department of Animal and Plant Sciences University of Sheffield Sheffield S10 2TN UK; ^2^ Durrell Institute of Conservation and Ecology (DICE) School of Anthropology and Conservation University of Kent Canterbury Kent CT2 7NR UK; ^3^ Environment and Sustainability Institute University of Exeter Penryn Cornwall TR10 9FE UK

**Keywords:** ecosystem services, food security, greenspace, grow your own, organic carbon, sustainable agriculture

## Abstract

1. Modern agriculture, in seeking to maximize yields to meet growing global food demand, has caused loss of soil organic carbon (SOC) and compaction, impairing critical regulating and supporting ecosystem services upon which humans also depend. Own‐growing makes an important contribution to food security in urban areas globally, but its effects on soil qualities that underpin ecosystem service provision are currently unknown.
2. We compared the main indicators of soil quality; SOC storage, total nitrogen (TN), C : N ratio and bulk density (BD) in urban allotments to soils from the surrounding agricultural region, and between the allotments and other urban greenspaces in a typical UK city. A questionnaire was used to investigate allotment management practices that influence soil properties.
3. Allotment soils had 32% higher SOC concentrations and 36% higher C : N ratios than pastures and arable fields and 25% higher TN and 10% lower BD than arable soils.
4. There was no significant difference between SOC concentration in allotments and urban non‐domestic greenspaces, but it was higher in domestic gardens beneath woody vegetation. Allotment soil C : N ratio exceeded that in non‐domestic greenspaces, but was lower than that in garden soil.
5. Three‐quarters of surveyed allotment plot holders added manure, 95% composted biomass on‐site, and many added organic‐based fertilizers and commercial composts. This may explain the maintenance of SOC, C : N ratios, TN and low BD, which are positively associated with soil functioning.
6. *Synthesis and applications*. Maintenance and protection of the quality of our soil resource is essential for sustainable food production and for regulating and supporting ecosystem services upon which we depend. Our study establishes, for the first time, that small‐scale urban food production can occur without the penalty of soil degradation seen in conventional agriculture, and maintains the high soil quality seen in urban greenspaces. Given the involvement of over 800 million people in urban agriculture globally, and its important contribution to food security, our findings suggest that to better protect soil functions, local, national and international urban planning and policy making should promote more urban own‐growing in preference to further intensification of conventional agriculture to meet increasing food demand.

## Abstract 2

Maintenance and protection of the quality of our soil resource is essential for sustainable food production and for regulating and supporting ecosystem services upon which we depend. Our study establishes, for the first time, that small‐scale urban food production can occur without the penalty of soil degradation seen in conventional agriculture, and maintains the high soil quality seen in urban greenspaces. Given the involvement of over 800 million people in urban agriculture globally, and its important contribution to food security, our findings suggest that to better protect soil functions, local, national and international urban planning and policy making should promote more urban own‐growing in preference to further intensification of conventional agriculture to meet increasing food demand.

## Introduction

Agriculture, at all scales of production, is dependent on the natural capital of soils which yield a flow of services upon which humans depend, not only for food, fibre and biomass production, but also for other ecosystem services such as provision of fresh water, regulation of nutrient cycling, flood mitigation, water purification, carbon sequestration and climate regulation (Kibblewhite, Ritz & Swift 2008; Haygarth & Ritz 2009; Dominati, Patterson & Mackay 2010; Robinson *et al*. 2013). During the 20th century, the rising demand for food globally was met by conversion of natural and semi‐natural habitats into agricultural land, and the intensification of farming methods, including mechanization and use of synthetic fertilizers (Robinson & Sutherland 2002; Haygarth & Ritz 2009). However, intensification of agriculture has depleted the natural capital of soil organic carbon (SOC) and nutrients resulting in serious losses of regulating and supporting ecosystem services (Franzluebbers 2002). These include impaired water and nutrient holding capacity, reduced pollutant immobilization and water filtration, loss of soil aggregates and strength (Watts & Dexter 1997) leading to increased erosion, CO_2_ release to the atmosphere and eutrophication of aquatic ecosystems (Robinson & Sutherland 2002; Loveland & Webb 2003; Dominati, Patterson & Mackay 2010; Robinson *et al*. 2013). Loss of organic matter (OM) content is of particular concern for food security as yields of staple cereal crops typically increase linearly with SOC concentration (Lal 2010).

One of the greatest challenges now facing humanity is to improve the sustainability of agriculture and reduce its environmental impact, whilst also meeting the food demands of the growing global population, which exceeds 7 billion (DEFRA 2010; Godfray *et al*. 2010). A crucial goal in agricultural sustainability is to reverse the historic losses of SOC from farmland and to increase soil C : N ratios which are important controls on nutrient cycle regulation (Robinson *et al*. 2013). High C : N‐rich soil amendments are particularly important in reducing the risk of N leaching from soils (Dungait *et al*. 2012).

Concurrent with the intensification of agriculture has been rapid urbanization; over half of the world's population is now residing in cities and towns (UN 2008). Indeed, urban areas are increasing in areal extent faster than any other land use (Hansen *et al*. 2005), a trend set to continue as the proportion of people living in cities and towns rises to 70% by 2050 (UN 2008). This land‐use change is further exacerbated by the expansion of urban areas outpacing population growth, particularly in developed regions such as Europe (EEA 2006). These dynamics bring about a number of significant challenges. Of increasing concern is the food security of urban inhabitants as they become physically more detached from primary food production (Howe & Wheeler 1999).

However, an estimated 800 million people currently practise some form of urban food production globally, with much borne out of necessity for subsistence in the developing world (Lee‐Smith 2010). Urban horticulture operates over spatial scales ranging from potted plants, to vegetable plots in gardens, to allotments, community gardens and city farms (Howe & Wheeler 1999). In Europe, allotments are a common feature of urban areas and in areal extent are often the main areas of own‐grown food production. In the UK, there are *c*. 330 000 allotment plots, and a standard plot is 250 m^2^, giving a total area nationally likely to be >8000 ha (Crouch & Ward 1997). Allotments represent a unique type of greenspace, designated specifically for food production (van den Berg *et al*. 2010). Peak allotment provision in the UK occurred during the First and Second World Wars (Crouch & Ward 1997; Martin & Marsden 1999), and during the latter, allotments and gardens provided *c*. 10% of food consumed in the UK because of the ‘Dig for Victory’ campaign whilst comprising <1% of the area of arable cultivation (Crouch & Ward 1997; Keep 2009).

After a post‐war decline in own‐growing and associated decrease in plot provision, there has been a resurgence in UK allotment demand reflected in increased waiting lists over the past 17 years, with over 90 000 people now waiting for a plot (Campbell & Campbell 2011). The increase in interest in agriculture is not confined to the UK, for example own‐growing in the USA has risen (Viljoen & Bohn 2012) as a result of a recognition of the importance of provision of healthy food, particularly to disadvantaged neighbourhoods in combination with the availability of ‘vacant lots’ within urban areas (Grewel & Grewel 2012). Amongst scientists, policymakers, the media and public, there is increasing awareness of the multiple benefits of ‘own‐growing’ including access to nutritious fresh produce, stress relief, improved psychological well‐being and physical fitness (Martin & Marsden 1999; Leake, Adam‐Bradford & Rigby 2009; van den Berg *et al*. 2010; Kortright & Wakefield 2011). The UK government £30 million Healthy Towns Initiative launched in 2008 funded projects aimed at increasing participation in own‐growing to promote healthier lifestyles and tackle the problem of sedentary behaviour, low consumption of fresh fruit and vegetables, and obesity. Other motivations for own‐growing include more sustainable living in response to threats from climate change, peak oil and unsustainable food production systems (Hopkins 2008), widespread concerns about chemical residues of pesticides in conventional agriculture, genetically modified crops and ‘food miles’. More recently, the increase in own‐growing has been attributed to rising global food prices (DEFRA 2010).

Soils in urban greenspaces have recently been shown to make an important contribution to provision of ecosystem goods and services especially in holding large stocks of SOC (Pouyat, Yesilonis & Nowak 2006; Churkina, Brown & Keoleian 2010; Edmondson *et al*. 2011, 2012, 2014). However, we currently know nothing about how soil management for own‐growing in allotments impacts on the main soil quality indicators. Do these soils suffer significant depletion in SOC and nitrogen stocks compared to other urban greenspaces, as might be expected on the basis of the effects of cultivation seen in conventional agriculture? Are higher SOC stocks maintained under perennial woody fruit bushes and trees, where soil may be less disturbed, compared to frequently dug ground used for annual herbaceous crops?

In this paper, we investigate topsoil properties at allotment sites across an entire mid‐sized UK city, including SOC concentration, total nitrogen (TN) concentration, C : N ratio and soil bulk density (BD), and compare them to urban domestic gardens, non‐domestic greenspace and regional agricultural soils. The comparisons with other urban greenspace soils were made to determine whether allotment cultivation significantly impacts urban soil quality within the same city on the same soil types, and these other greenspaces provide a ‘control’ for soil properties that are affected by the urban environment such as air pollutants. The soil properties were selected as they are positively associated with regulating and supporting ecosystem services (Franzluebbers 2002) and can be directly managed for ecosystem service provision (Kibblewhite, Ritz & Swift 2008; Dominati, Patterson & Mackay 2010). SOC is particularly important as it has a direct positive influence on both ecosystem function including water and nutrient holding capacity, and crop growth and C : N ratio is one of the major controls of both N and C cycling in the soil (Powlson *et al*. 2011; Dungait *et al*. 2012). BD is a direct measure of soil pore space, which provides an indication of the ability of soil store water and the rate of storm water infiltration (Lal 2007; Dominati, Patterson & Mackay 2010).

Using a questionnaire, we examine plot management practices which may influence soil quality in allotments including the prevalence of on‐site composting; inputs of manure, fertilizer and commercial compost; and the burning or removal of OM for disposal off‐site.

We hypothesize that (i) intensively managed urban allotments will maintain higher soil quality, as indicated by the above parameters when compared to regional agricultural soil, and (ii) cultivation on allotments will negatively affect soil properties in comparison with other types of urban greenspace, to a greater extent in beds used for annual crops than under woody fruit bushes and trees.

## Materials and methods

### Study Area

**Figure 1:**
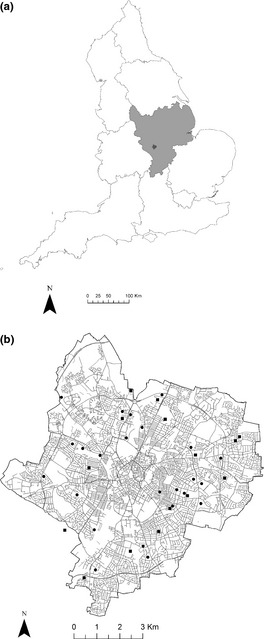
(a) The geographical location of the East Midlands within England and our study city, Leicester, and (b) the position of allotments within Leicester. Square symbols represent allotment sites sampled; circular symbols are unvisited allotment sites.

Our study focussed on Leicester, a mid‐sized UK city in the East Midlands of England (52°38′N, 1°08W), covering an area of *c*. 73 km^2^ (defined by the unitary authority boundary), with a human population of *c*. 330,000 (Leicester City Council 2013; Fig. 1a). The region experiences a temperate climate, receiving 606 mm of precipitation annually and average annual daily minimum and maximum temperatures of 5·8 and 13·5 °C, respectively (Met Office 2009). More than 75% of land in the East Midlands is agricultural, of which arable farming is dominant (Rural Business Research 2012). Soils within the city are deep clays, deep loam and seasonally wet deep clays and loam, according to the National Soil Map for England and Wales produced by Cranfield University. The main soil series in the city and its agricultural hinterland are Hanslope, Whimple, Salop, Beccles 3, Ragdale and Fladbury 1.

Allotment provision in Leicester peaked in the 1930s, with one household in three renting a plot (Crouch & Ward 1997). Today, the city has 46 allotment sites (Fig. 1b), 45 of which are owned by Leicester City Council with 3200 individual plots (Leicester City Council 2012) that in total cover *c*. 2% of the cities greenspace. Within the city, greenspace constitutes 56% of the total area with 32% managed on a small scale privately in domestic gardens, and the remaining 68% is non‐domestic greenspace generally managed on a large scale by Leicester City Council or large institutions.

### Soil Survey

Fifteen allotment sites were selected to provide representative samples from across the city (Fig. 1b), with permission obtained to sample from 27 plots. Where permission was granted, the cultivation on the allotment plot was assessed, specifically the presence of annual herbaceous vegetable crops (which generally constitute the largest cultivated area) and perennial fruit bushes and trees. In all plots, duplicate soil cores were taken under annual vegetable crops and, where available, another duplicate set of samples were taken under woody fruit bushes or trees. Samples were taken from the topsoil layer in two depth increments (0–7 cm and 7–14 cm), using specialist corer that removes undisturbed soil samples for BD analysis (Edmondson *et al*. 2011).

Sample locations for soils in other urban greenspaces were generated in a GIS using two high spatial resolution data sets. The first, LandBase was produced by Infoterra (http://geosurveysolutions.com/landbase; accessed April 2014) and categorized land cover within the city into eight different classes (inland water, bare ground, artificial surface, buildings, herbaceous vegetation, shrubs, tall shrubs and trees; Davies *et al*. 2013). The LandBase data set used high‐resolution LiDar data to stratify vegetation by height. This data set determined the extent of greenspace within the city and the extent of the different vegetation land‐cover classes used in this study (herbaceous vegetation and a combined shrubs, tall shrubs and tree category). The second data set, MasterMap, provided by Ordnance Survey (http://www.ordnancesurvey.co.uk/business-and-government/products/mastermap-products.html), was used to split the two land‐cover classes by land use into domestic gardens and non‐domestic greenspace. Random sample points were generated within the GIS for the different non‐domestic greenspace land‐cover categories and, at each, four replicate soil samples were taken at the two depth intervals. The sampling strategy for domestic gardens used a street layer created in the GIS and 45 roads were selected at random. Each of these roads was visited and, if there were residential properties present and authorization from a householder was granted, soil cores were taken from the back garden. In domestic gardens, cores were extracted from herbaceous areas within the garden and/or within the vicinity of shrubs and trees (where gardens contained both land‐cover classes cores were taken beneath both herbaceous vegetation and shrubs and trees). In total, a further 136 sites were sampled within the urban greenspace of the city. Similarly, soil samples were taken from randomly selected agricultural sites (arable *n* = 16; pasture *n* = 12), within a 7·5‐km buffer zone around the unitary authority boundary of Leicester (see Table S1 in Supporting Information for sample site GPS coordinates).

### Soil Sample Preparation and Analysis

Soils were dried at 105 °C for 24 h, weighed, ball‐milled to homogenize and passed through a 1‐mm sieve. Material >1 mm was weighed and then removed from the soil total weight (Edmondson *et al*. 2011). Soil BD was converted to g cm^−3^. The homogenized samples were analysed for C and TN in an elemental analyser (VarioEL Cube; Isoprime, Hanau, Germany; Edmondson *et al*. 2012). SOC density (mg cm^−3^) was calculated for each individual sample using SOC concentration (mg g^−1^) and BD (g cm^−3^) following the approach of Edmondson *et al*. (2012).

### Allotment Questionnaire

All allotment holders present at the time of the site visit were asked to complete a questionnaire (see Appendix S1, Supporting Information) about plot management. The questionnaire assessed the length of time the plot had been held by the present person; types of OM added; types of fertilizer used; OM burning or removal from the plot. In total, 75 plot holders, including those where soil was sampled, answered the questionnaire.

### Statistical Analysis

The effects of urban allotment vs. agricultural land use (arable and pasture) on soil properties, including effects in relation to soil depth, were analysed using two‐way anova. The effect of urban land use (allotment, domestic garden and non‐domestic greenspace), soil depth and vegetation cover (tree and shrub or herbaceous) on soil properties was analysed using three‐way anova. The Tukey *post hoc* test compared differences (*P *<* *0·05) between means (Zar 1999). All data were checked for homogeneity of variance and normality prior to analysis and, where necessary, were transformed. Analyses were conducted in pasw Statistics 18.

## Results

### Allotment Management

The length of time the 75 allotment holders had managed their plots ranged from <1 to 50 years, with a median duration of 5 years and with 16% of respondents having held their plots for more than 15 years. In total, 95% of the respondents composted on their plot, with 73% adding household fruit and vegetable waste to their allotment compost. Nearly half of the respondents added commercial compost to their plot soils (Table 1). Three‐quarters of respondents added manure and a similar proportion bought other fertilizers (Table 1). These included general purpose mineral fertilizer, chicken manure and fish blood and bone, with 53%, 42% and 27% of respondents using these products, respectively, and a minority using tomato feed, liquid seaweed and lime.

**Table 1:** Responses of allotment holders to a questionnaire focused on plot management. Number of survey respondents = 75

Questions	Yes (%)	No (%)	No answer (%)
Compost production on allotments			
Do you compost your waste allotment material?	95	5	0
Do you compost household vegetable matter on your allotment?	72	27	1
Inputs to allotments			
Do you add commercial compost to your allotment?	45	50	5
Do you add manure to your allotment?	75	20	5
Do you add any fertilizer to your allotment?	73	21	5
Removals from allotments			
Do you burn material from your allotment?	68	28	4
Do you remove any tree, shrub or hedge trimmings from you allotment?	17	63	20
Do you remove any autumn leaves from your allotment?	8	72	20

Biomass that is slow to compost, including tree, shrub or hedge trimmings, sweetcorn stalks and brassica roots, together with diseased plants and noxious weeds, was burnt on‐site by 68% of respondents (Table 1). A smaller proportion of allotment holders acknowledge removing these kinds of wastes, and autumn leaves, from their plots for disposal elsewhere (Table 1).

### The Effect of Own‐Growing vs. Conventional Agriculture on Soil Properties

**Figure 2:**
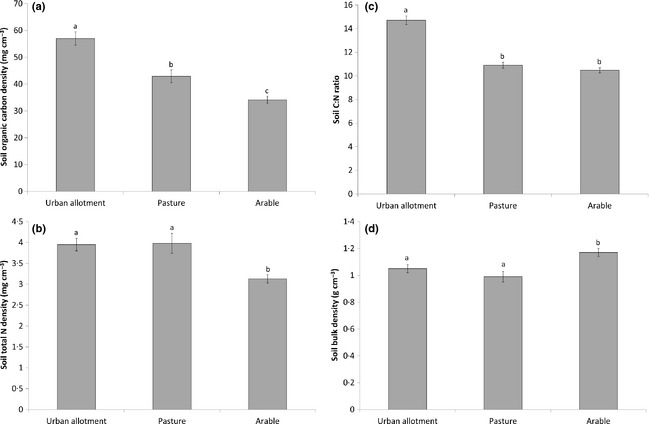
(a) Mean soil organic carbon density; (b) soil nitrogen density; (c) soil C : N ratio; (d) soil bulk density in urban allotment and agricultural soils. Error bars are ±1 standard error; letters show significant differences between land uses (Tukey's test *P *<* *0·05).

Soil organic carbon density (mg cm^−3^) was significantly higher in allotments compared to soils from surrounding agricultural land, with arable land most seriously depleted in SOC having 65% lower concentrations than allotment soils (Fig. 2a). Soil TN density (mg cm^−3^) was also significantly reduced in arable fields, with 25% greater TN densities in both pasture and allotment soils (Fig. 2b). As with SOC density, soil C : N ratio was 36% greater in allotments than in conventional agriculture but, in this case, there was no significant difference between pasture and arable fields (Fig. 2c). Soil BD was 15% lower in allotments and pasture compared to arable fields (Fig. 2d). There was no effect of soil depth on SOC density, C : N ratio or BD, or any interaction between land use and depth (Table 2). Soil TN density declined significantly with depth (Table 2), driven by the responses of pasture and arable soils only (see Fig. S1, Supporting Information).

**Table 2:** Two‐way anova testing the effects of land use (urban allotments vs. intensive agriculture) and depth (0–7 cm, 7–14 cm) on soil organic carbon density, C : N ratio and soil bulk density. Numbers in bold indicate a significant effect

Transformation	Factor	d.f.	*F*	*P* value
Soil organic carbon density (mg cm^−3^)				
Log_10_	Land use	2,90	**32·294**	**<0·001**
	Soil depth	1,90	2·297	0·112
	Land use × soil depth	2,90	1·073	0·378
Soil nitrogen density (mg cm^−3^)				
Log_10_	Land use	2,89	**7·721**	**0·001**
	Soil depth	1,89	**4·060**	**0·047**
	Land use × soil depth	2,89	1·792	0·173
Soil C : N ratio				
Log_10_	Land use	2,91	**66·202**	**<0·001**
	Soil depth	1,91	0·123	0·727
	Land use × soil depth	2,91	0·172	0·842
Soil fine earth bulk density (g cm^−3^)				
	Land use	2,91	**6·479**	**0·002**
	Soil depth	1,91	0·004	0·947
	Land use × soil depth	2,91	1·031	0·361

### The Effect of Allotments vs. Other Urban Greenspace Land Uses on Soil Properties

**Figure 3:**
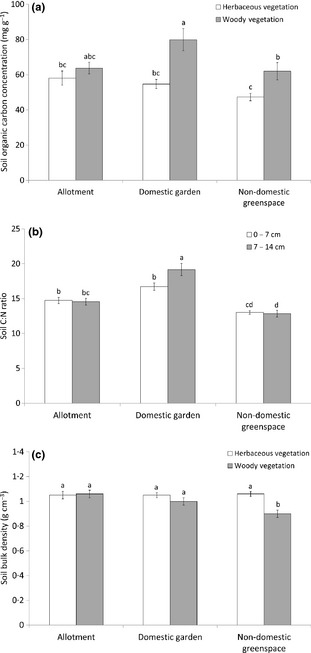
(a) Mean soil organic carbon concentration beneath woody and herbaceous vegetation; (b) C : N ratio at 7 and 14 cm soil depth; (c) soil bulk density beneath woody and herbaceous vegetation in three urban greenspace land‐use types. Error bars are ±1 standard error; letters show significant differences between land uses (Tukey's test *P *<* *0·05).

Soil organic carbon concentration (mg g^−1^) was significantly higher in gardens beneath woody vegetation than in all herbaceous vegetation (at least 37%) and 25% greater than under woody vegetation in non‐domestic land (Fig. 3a; Table 3). There was no difference in SOC concentration between soils beneath woody and herbaceous vegetation on allotments and beneath herbaceous vegetation throughout land‐use types. Consequently, there was a significant interaction between land use and vegetation on SOC concentration (Table 3).

**Table 3:** Three‐way anova testing effects of urban land use (allotment, domestic garden and non‐domestic), vegetation type (herbaceous or tree and shrub) and depth (0–7 cm, 7–14 cm) on soil organic carbon concentration, C : N ratio and soil bulk density. Numbers in bold indicate a significant effect.

Transformation	Factor	d.f.	*F*	*P* value
Soil organic carbon concentration (mg g^−1^)	Land use	2,283	**4·579**	**0·011**
	Vegetation type	1,283	**10·271**	**0·002**
	Soil depth	1,283	**8·916**	**0·003**
	Land use × vegetation	2,283	**3·193**	**0·043**
	Land use × depth	2,283	0·483	0·618
	Vegetation × depth	1,283	0·173	0·678
	Land use × vegetation × soil depth	2,283	0·656	0·520
Soil C : N ratio				
Log_10_	Land use	2,282	**76·216**	**<0·001**
	Vegetation type	1,282	**8·237**	**0·004**
	Soil depth	1,282	0·699	0·404
	Land use × vegetation	2,282	2·911	0·056
	Land use × depth	2,282	**3·540**	**0·030**
	Vegetation × depth	1,282	0·736	0·392
	Land use × vegetation × soil depth	2,282	1·485	0·228
Soil bulk density (g cm^−3^)				
	Land use	2,268	2·361	0·096
	Vegetation type	1,268	**11·304**	**<0·001**
	Soil depth	1,268	**12·408**	**<0·001**
	Land use × vegetation	2,268	**3·945**	**0·020**
	Land use × depth	2,268	0·241	0·786
	Vegetation × depth	1,268	0·251	0·617
	Land use × vegetation × soil depth	2,268	0·195	0·823

There was a significant land‐use effect on soil C : N ratio (Table 3), with lowest values in non‐domestic greenspaces and higher values in gardens between 7–14 cm depth, compared to allotments (Fig. 3b). There was also a significant effect of land‐cover type on C : N ratio and there was a significant interaction between land use and soil depth (Table 3, Fig. 3b).

Greenspace land use had no effect on soil BD (Table 3). There was significantly lower soil BD beneath trees and shrubs compared to herbaceous vegetation. This effect was driven by differences arising in the non‐domestic greenspaces but not in allotments resulting in a significant interaction between vegetation and land use (Fig. 3c, Table 3). Soil BD beneath woody vegetation in non‐domestic greenspace was 17% lower than soils beneath herbaceous vegetation throughout the land‐use categories and at least 11% lower than under woody vegetation in domestic gardens or allotments (Fig. 3c). BD significantly increased with depth across all urban greenspace land uses for both herbaceous and woody vegetation (Table 3).

## Discussion

### The Properties of Allotment, Domestic Garden and Non‐Domestic Greenspace Soils

Until recently, in the absence of city‐scale sampling, soils in urban areas have often been represented as functionally degraded, low in OM and compacted. This follows from research on urban soils mainly focussed on highly altered and disturbed areas, often associated with land redevelopment generating ‘technosols’ whose formation and functioning are the result of anthropogenic activities (Lehmann & Stahr 2007), but are not representative of typical urban soils. Furthermore, it has often been assumed that urban centres are devoid of functional soil, for example in the UK national SOC inventory (Bradley *et al*. 2005) it has been assumed that city centres contain no SOC, and soils in suburban areas hold half of the SOC concentration of regional pasture soils. Until recently, these assumptions remained untested. Empirical evidence has now challenged these assumptions on two fronts. First, high spatial resolution GIS has revealed the extent of urban greenspace, even in built‐up city centres, is greater than previously recognized, for example Leicester contains 56% greenspace, including a very large number of small patches undetected by lower spatial resolution data sets (Davies *et al*. 2013). Secondly, measurements of urban SOC stocks (Pouyat, Yesilonis & Nowak 2006; Churkina, Brown & Keoleian 2010), which include some studies at the citywide scale, have revealed SOC concentrations and soil BD values comparable to those in semi‐natural ecosystems (Edmondson *et al*. 2011, 2012). In the present paper, we extend these findings to show that across the suite of soil properties measured within allotments, soil quality was consistently high, compared to soils from the surrounding agricultural region, and against English national data (Carey *et al*. 2008). Whilst arable agriculture is the dominant land use in the Leicester region, allotment soil properties compare favourably to those found in semi‐natural habitats. For example, when compared to English lowland woodland soils and grasslands of neutral pH, SOC storage was *c*. 1 kg m^−2^ greater and TN storage was similar (0·55, 0·61 and 0·53 kg m^−2^ for allotments, neutral grasslands and lowland woodlands, respectively; Carey *et al*. 2008). Soil BD was slightly higher in allotments when compared to these two semi‐natural habitats ranging from 1·1 g cm^−3^ in allotments, 0·9 g cm^−3^ in neutral grasslands, down to 0·8 g cm^−3^ in lowland woodland soils (Carey *et al*. 2008). Soil C : N ratio was considerably higher in lowland woodlands (17·7) than in allotment (14·7) and neutral grassland soils (14·1; Carey *et al*. 2008).

The modest differences between soil properties in allotments compared to other urban greenspaces, and the indicators of high soil quality in all these greenspaces, affirm the new paradigm of typical soils in urban areas being of high ecological and ecosystem service value (Edmondson *et al*. 2011; Edmondson *et al*. 2012); however, research must be conducted in cities world‐wide to further support these findings. This parallels the recent paradigm shift in recognition of the importance of urban areas for biodiversity. The ‘urban desert’ myth (Braat & ten Brink 2008) was overturned by systematic data collection across multiple UK cities, revealing that native and alien species richness and habitat diversity exceeded that in the wider countryside on a unit area basis (Loram, Warren & Gaston 2008; Loram *et al*. 2008).

It is clear that urban soils not only play very important roles in the delivery of supporting and regulating ecosystem services such as carbon, water and nutrient storage, but also in the provisioning service of food production. Most supporting ecosystem services depend on SOC (Powlson *et al*. 2011), so the finding that allotment soils contain more SOC than the soils in non‐domestic greenspaces is important. Although garden soils had slightly higher SOC than in allotments, this was strongly driven by values beneath trees. Extra accumulation of SOC under trees and shrubs in gardens is likely to occur due to reduced disturbance and increased leaf litter, composts and mulch inputs (Osmond & Hardy 2004), compared to other greenspace soils. In addition, as the city of Leicester expanded over agricultural land, albeit before the advent of modern agriculture that intensified SOC loss, it is more likely that SOC storage has increased in gardens under trees and shrubs, than allotment soils have significantly lost SOC compared to the pre‐urbanization concentrations in agricultural land. Furthermore, the lower SOC concentrations in non‐domestic greenspaces compared to allotment soils suggest that the additional carbon inputs to allotments, especially manure and compost, are important in maintaining or increasing SOC storage.

The absence of beneficial effects of woody vegetation on SOC in allotment soils, in contrast to the effects seen in domestic and non‐domestic greenspaces, is probably explained by the woody plants on allotments being of low stature and biomass. They comprise woody‐stemmed fruit bushes, and small fruit trees, often grafted onto dwarfing rooting stocks and will not be very long established as the median duration of plot holding was 5 years. Many local authorities discourage or forbid cultivation of fruit trees and bushes on allotments and require removal of such plants before plots are allocated to new tenants, so old established fruit trees are rare on allotments compared to gardens.

### Comparison of Allotment and Agricultural Soils

The remarkable contrast in soil quality indicators (higher SOC, C : N, TN and lower BD) between allotments and arable fields reveals the effectiveness of management achieved by own‐growers. Furthermore, it demonstrates the extent to which modern agricultural practices have degraded soil natural capital – which has profound implications for the loss of ecosystem service provision (Loveland & Webb 2003; Lal 2004), including reduced structural stability, water and nutrient holding capacity and impaired regulation of N mineralization and supply to plants (Quinton *et al*. 2010; Dungait *et al*. 2012). In terms of provisioning ecosystem services by own‐growing in allotments, both the historical records of production during the world wars and more recent UK trials conducted by the Royal Horticultural Society and ‘Which?’ Magazine showed fruit and vegetable yields of 31–40 t ha^−1^ year^−1^ (Tomkins 2006), 4–11 times the productivity of the major agricultural crops in the Leicestershire region (DEFRA 2013). Importantly, depletion of SOC in conventional agricultural fields is now thought to be an important factor constraining productivity as many arable soils have suboptimal concentrations (Lal 2010).

However, our data revealing the maintenance of soil quality in allotments indicate that benefits obtained from ecosystem services do not necessarily have to be traded off against each other in the manner currently seen in conventional agriculture. Indeed, allotments not only provide yields rarely matched by commercial horticulture (Tomkins 2006), but simultaneously provide exceptionally high delivery of a wide portfolio of other ecosystem services. These include cultural services such as aesthetic value, together with physical and psychological benefits (Leake, Adam‐Bradford & Rigby 2009; Kortright & Wakefield 2011). Most importantly, we show that the soil quality indicators underpinning the delivery of supporting ecosystem services are not compromised by the delivery of provisioning and cultural ecosystem services for which allotments are most valued. Urban agriculture is already a well‐established management practice in urban greenspaces globally, and interest in own‐growing is increasing. Consequently, policy and planning at local, national and international scales should seek to capitalize on this resurgence in interest and further encourage urban agriculture as a means to improve food security within cities and towns, as this can deliver additional food without compromising soil quality. This is in contrast to conventional agriculture in which intensification of production generally leads to the loss of soil OM and quality (Lal 2010).

The maintenance of this multifunctionality of allotments rests on substantial inputs of OM and nutrients to these soils including manures, and own‐produced composts, all of which have been shown to increase SOC and TN concentrations (Lal 2004, 2008). However, it is important to recognize that many of these inputs involve a ‘subsidy’ from agriculture and fisheries such as cow and chicken manure, commercial composts and purchased vegetable and fruit waste composted on allotments. This subsidy from agriculture may be justified by higher yields obtained by own‐growing, but tempers any claim that allotment cultivation is completely sustainable. In addition, 55% of respondents used a synthetic fertilizer, many of which are derived from, or produced using, petrochemicals. The increasing use of large‐scale composting of putrescible household wastes by local councils instead of landfilling opens the possibility for reduced dependence on agricultural products and greater sustainability if these composts can be substituted on allotments for other organic carbon and nutrient sources.

We found evidence of both environmentally favourable and unfavourable management practices on allotments. For example, compost heaps are recognized as an indicator of urban biodiversity and are of particular importance as habitats for invertebrate communities (DEFRA 2003). However, other practices may be less favourable, and 68% of respondents reported burning material, a frequency far greater than in a recent survey reporting 15% of gardeners use bonfires to manage waste (Loram *et al*. 2011). Whilst application of ash and char from these fires onto allotment soils could further improve soil quality, as the biochars produced are a highly stable form of OC (Lehmann 2007), bonfires are detrimental to air quality and a risk to human health in highly populated urban areas.

### Conclusions

This research demonstrates that own‐growing in urban allotments, in contrast to arable crop production, does not trade off the soil quality measures that are positively associated with regulating and supporting ecosystem services, in order to deliver provisioning ecosystem services. Typical urban soils are shown to be comparable to semi‐natural ecosystems and of considerably better quality than agricultural soils, and this is maintained under cultivation in allotments, which receive regular organic inputs from manures and composts. Allotment cultivation may provide a model for understanding management systems for sustainably delivering multiple ecosystem services without the provision of one type of service compromising the delivery of another. Further work is now required to quantify the ecosystem services provided by allotments, the potential and actual yield of crops in urban environments, and the area currently under cultivation. For urban allotment cultivation to be more sustainable, efforts should be made to replace OM and nutrient inputs derived directly from agriculture with those derived from composting putrescible wastes in cities. Our findings lend additional support to the view that own‐growing provides multiple human and environmental benefits and has a role to play in more sustainable living in urban areas.

## Supporting information


**Fig. S1**. Mean soil total nitrogen density in urban allotments and agricultural soils at 0 ‐ 7 and 7 ‐ 14 cm depth.

**Table S1**. GPS coordinates of each soil sample site.

**Appendix S1**. Allotment questionnaire.
