# Supplementary material for: Urban cultivation in allotments maintains soil qualities adversely affected by conventional agriculture
Source: J Appl Ecol. 2014 Apr 24;51(4):880–9. doi: 10.1111/1365-2664.12254 (PMC4301088; doi:10.1111/1365-2664.12254)
Supplement: Supplementary file 1 — Fig. S1. Mean soil total nitrogen density in urban allotments and agricultural soils at 0 ‐ 7 and 7 ‐ 14 cm depth. [file JPE-51-880-s001.pdf]

Fig. S1: Mean soil nitrogen density in urban allotment and agricultural soils: 0 – 7 cm depth represented by white bars; 7 – 14 cm indicated by grey bars. Error bars are ±1 standard error.

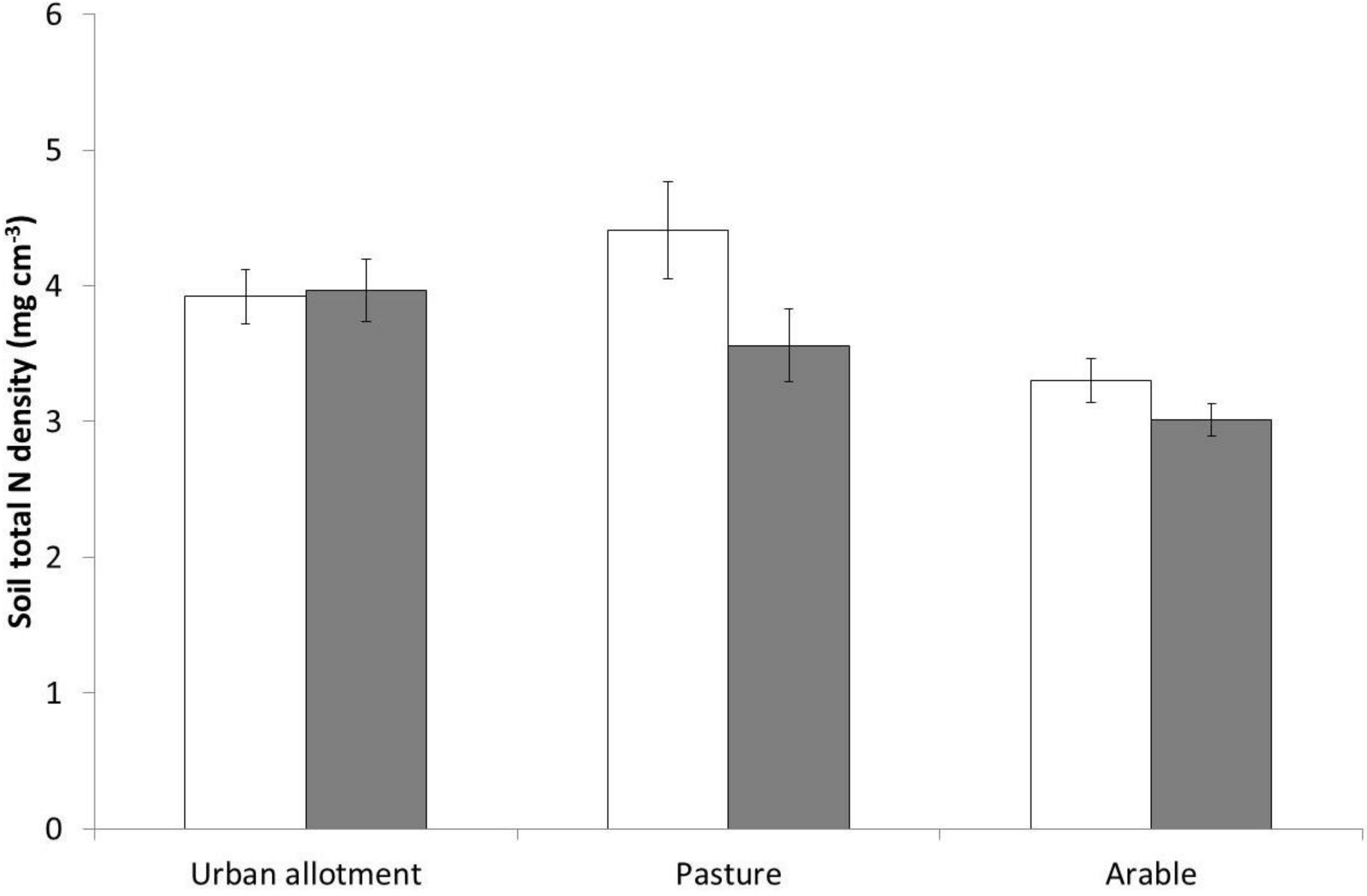

Jill L Edmondson, Zoe G. Davies, Kevin J. Gaston, Jonathan R. Leake. Urban cultivation in allotments maintains soil qualities adversely affected by conventional agriculture
