# Supplementary material for: Urban cultivation in allotments maintains soil qualities adversely affected by conventional agriculture
Source: J Appl Ecol. 2014 Apr 24;51(4):880–9. doi: 10.1111/1365-2664.12254 (PMC4301088; doi:10.1111/1365-2664.12254)
Supplement: Supplementary file 2 — Table S1. GPS coordinates of each soil sample site. [file JPE-51-880-s002.pdf]

Urban cultivation in allotments maintains soil qualities adversely affected by conventional agriculture

Jill L Edmondson, Zoe G. Davies, Kevin J. Gaston, Jonathan R. Leake

Table S1: GPS coordinates of each soil sample site

Land-use	Landcover	Latitude	Longitude
Agriculture	Arable	52.63	-1.22
Agriculture	Arable	52.67	-1.14
Agriculture	Arable	52.67	-1.13
Agriculture	Arable	52.69	-1.05
Agriculture	Arable	52.61	-1.21
Agriculture	Arable	52.57	-1.14
Agriculture	Arable	52.56	-1.22
Agriculture	Arable	52.55	-1.29
Agriculture	Arable	52.57	-1.26
Agriculture	Arable	52.60	-1.20
Agriculture	Arable	52.57	-0.95
Agriculture	Arable	52.56	-1.05
Agriculture	Arable	52.63	-1.30
Agriculture	Arable	52.58	-1.20
Agriculture	Arable	52.73	-1.15
Non-domestic greenspace	Herbaceous vegetation	52.60	-1.16
Non-domestic greenspace	Herbaceous vegetation	52.66	-1.15
Non-domestic greenspace	Herbaceous vegetation	52.61	-1.15
Non-domestic greenspace	Herbaceous vegetation	52.62	-1.12
Non-domestic greenspace	Herbaceous vegetation	52.62	-1.12
Non-domestic greenspace	Herbaceous vegetation	52.66	-1.06
Non-domestic greenspace	Herbaceous vegetation	52.62	-1.11
Non-domestic greenspace	Herbaceous vegetation	52.64	-1.13
Non-domestic greenspace	Herbaceous vegetation	52.59	-1.10
Non-domestic greenspace	Herbaceous vegetation	52.64	-1.16
Non-domestic greenspace	Herbaceous vegetation	52.65	-1.12
Non-domestic greenspace	Herbaceous vegetation	52.60	-1.10
Non-domestic greenspace	Herbaceous vegetation	52.60	-1.15
Non-domestic greenspace	Herbaceous vegetation	52.66	-1.09
Non-domestic greenspace	Herbaceous vegetation	52.59	-1.11
Non-domestic greenspace	Herbaceous vegetation	52.64	-1.18
Non-domestic greenspace	Herbaceous vegetation	52.66	-1.13
Non-domestic greenspace	Herbaceous vegetation	52.64	-1.16
Non-domestic greenspace	Herbaceous vegetation	52.62	-1.17
Non-domestic greenspace	Herbaceous vegetation	52.63	-1.17
Non-domestic greenspace	Herbaceous vegetation	52.64	-1.18
Non-domestic greenspace	Herbaceous vegetation	52.58	-1.14
Non-domestic greenspace	Herbaceous vegetation	52.64	-1.06
Non-domestic greenspace	Herbaceous vegetation	52.60	-1.13
Non-domestic greenspace	Herbaceous vegetation	52.60	-1.16
Non-domestic greenspace	Herbaceous vegetation	52.63	-1.05
Non-domestic greenspace	Herbaceous vegetation	52.63	-1.17
Non-domestic greenspace	Herbaceous vegetation	52.65	-1.08
Non-domestic greenspace	Herbaceous vegetation	52.62	-1.17
Non-domestic greenspace	Herbaceous vegetation	52.62	-1.11

Non-domestic greenspace	Herbaceous vegetation	52.62	-1.12
Non-domestic greenspace	Herbaceous vegetation	52.62	-1.17
Non-domestic greenspace	Herbaceous vegetation	52.68	-1.17
Domestic garden	Herbaceous vegetation	52.60	-1.10
Domestic garden	Herbaceous vegetation	52.65	-1.09
Domestic garden	Herbaceous vegetation	52.66	-1.16
Domestic garden	Herbaceous vegetation	52.62	-1.09
Domestic garden	Herbaceous vegetation	52.59	-1.15
Domestic garden	Herbaceous vegetation	52.62	-1.08
Domestic garden	Herbaceous vegetation	52.65	-1.04
Domestic garden	Herbaceous vegetation	52.62	-1.19
Domestic garden	Herbaceous vegetation	52.65	-1.16
Domestic garden	Herbaceous vegetation	52.63	-1.14
Domestic garden	Herbaceous vegetation	52.60	-1.13
Domestic garden	Herbaceous vegetation	52.65	-1.07
Domestic garden	Herbaceous vegetation	52.64	-1.14
Domestic garden	Herbaceous vegetation	52.67	-1.15
Domestic garden	Herbaceous vegetation	52.60	-1.11
Domestic garden	Herbaceous vegetation	52.68	-1.14
Domestic garden	Herbaceous vegetation	52.60	-1.13
Domestic garden	Herbaceous vegetation	52.63	-1.09
Domestic garden	Herbaceous vegetation	52.62	-1.09
Domestic garden	Herbaceous vegetation	52.62	-1.16
Domestic garden	Herbaceous vegetation	52.65	-1.09
Domestic garden	Herbaceous vegetation	52.61	-1.16
Domestic garden	Herbaceous vegetation	52.65	-1.16
Domestic garden	Herbaceous vegetation	52.64	-1.10
Domestic garden	Herbaceous vegetation	52.62	-1.16
Domestic garden	Herbaceous vegetation	52.63	-1.14
Domestic garden	Herbaceous vegetation	52.60	-1.10
Domestic garden	Herbaceous vegetation	52.66	-1.15
Domestic garden	Herbaceous vegetation	52.63	-1.14
Domestic garden	Herbaceous vegetation	52.67	-1.15
Allotment	Herbaceous vegetation	52.61	-1.17
Allotment	Herbaceous vegetation	52.66	-1.11
Allotment	Herbaceous vegetation	52.63	-1.15
Allotment	Herbaceous vegetation	52.66	-1.13
Allotment	Herbaceous vegetation	52.66	-1.13
Allotment	Herbaceous vegetation	52.66	-1.13
Allotment	Herbaceous vegetation	52.65	-1.13
Allotment	Herbaceous vegetation	52.65	-1.13
Allotment	Herbaceous vegetation	52.63	-1.07
Allotment	Herbaceous vegetation	52.60	-1.13
Allotment	Herbaceous vegetation	52.60	-1.13
Allotment	Herbaceous vegetation	52.61	-1.11
Allotment	Herbaceous vegetation	52.64	-1.06
Allotment	Herbaceous vegetation	52.62	-1.09
Allotment	Herbaceous vegetation	52.62	-1.10
Allotment	Herbaceous vegetation	52.62	-1.11
Allotment	Herbaceous vegetation	52.66	-1.13

Allotment	Herbaceous vegetation	52.62	-1.10
Allotment	Herbaceous vegetation	52.64	-1.09
Agriculture	Pasture	52.68	-1.24
Agriculture	Pasture	52.65	-1.16
Agriculture	Pasture	52.55	-1.17
Agriculture	Pasture	52.66	-1.07
Agriculture	Pasture	52.70	-1.12
Agriculture	Pasture	52.55	-1.07
Agriculture	Pasture	52.57	-1.12
Agriculture	Pasture	52.57	-1.08
Agriculture	Pasture	52.59	-0.98
Agriculture	Pasture	52.58	-0.94
Agriculture	Pasture	52.53	-1.13
Agriculture	Pasture	52.66	-1.04
Non-domestic greenspace	Woody vegetation	52.66	-1.15
Non-domestic greenspace	Woody vegetation	52.62	-1.07
Non-domestic greenspace	Woody vegetation	52.59	-1.14
Non-domestic greenspace	Woody vegetation	52.66	-1.16
Non-domestic greenspace	Woody vegetation	52.63	-1.10
Non-domestic greenspace	Woody vegetation	52.61	-1.09
Non-domestic greenspace	Woody vegetation	52.63	-1.17
Non-domestic greenspace	Woody vegetation	52.65	-1.17
Non-domestic greenspace	Woody vegetation	52.62	-1.17
Non-domestic greenspace	Woody vegetation	52.63	-1.17
Non-domestic greenspace	Woody vegetation	52.68	-1.17
Non-domestic greenspace	Woody vegetation	52.63	-1.05
Non-domestic greenspace	Woody vegetation	52.58	-1.14
Non-domestic greenspace	Woody vegetation	52.63	-1.20
Non-domestic greenspace	Woody vegetation	52.63	-1.11
Non-domestic greenspace	Woody vegetation	52.65	-1.08
Non-domestic greenspace	Woody vegetation	52.62	-1.17
Non-domestic greenspace	Woody vegetation	52.63	-1.17
Non-domestic greenspace	Woody vegetation	52.61	-1.11
Non-domestic greenspace	Woody vegetation	52.61	-1.13
Non-domestic greenspace	Woody vegetation	52.65	-1.15
Non-domestic greenspace	Woody vegetation	52.60	-1.15
Non-domestic greenspace	Woody vegetation	52.60	-1.11
Non-domestic greenspace	Woody vegetation	52.66	-1.16
Non-domestic greenspace	Woody vegetation	52.62	-1.07
Non-domestic greenspace	Woody vegetation	52.64	-1.16
Non-domestic greenspace	Woody vegetation	52.64	-1.13
Non-domestic greenspace	Woody vegetation	52.64	-1.12
Non-domestic greenspace	Woody vegetation	52.63	-1.15
Non-domestic greenspace	Woody vegetation	52.62	-1.17
Non-domestic greenspace	Woody vegetation	52.66	-1.16
Non-domestic greenspace	Woody vegetation	52.64	-1.16
Non-domestic greenspace	Woody vegetation	52.62	-1.18
Non-domestic greenspace	Woody vegetation	52.61	-1.14
Non-domestic greenspace	Woody vegetation	52.60	-1.15
Non-domestic greenspace	Woody vegetation	52.62	-1.18

Non-domestic greenspace	Woody vegetation	52.60	-1.13
Non-domestic greenspace	Woody vegetation	52.65	-1.07
Non-domestic greenspace	Woody vegetation	52.67	-1.17
Non-domestic greenspace	Woody vegetation	52.64	-1.13
Non-domestic greenspace	Woody vegetation	52.62	-1.17
Non-domestic greenspace	Woody vegetation	52.65	-1.07
Non-domestic greenspace	Woody vegetation	52.64	-1.12
Non-domestic greenspace	Woody vegetation	52.58	-1.15
Non-domestic greenspace	Woody vegetation	52.67	-1.17
Non-domestic greenspace	Woody vegetation	52.66	-1.12
Non-domestic greenspace	Woody vegetation	52.64	-1.16
Non-domestic greenspace	Woody vegetation	52.63	-1.14
Non-domestic greenspace	Woody vegetation	52.62	-1.14
Non-domestic greenspace	Woody vegetation	52.62	-1.17
Non-domestic greenspace	Woody vegetation	52.61	-1.15
Domestic garden	Woody vegetation	52.60	-1.10
Domestic garden	Woody vegetation	52.64	-1.08
Domestic garden	Woody vegetation	52.63	-1.09
Domestic garden	Woody vegetation	52.62	-1.07
Domestic garden	Woody vegetation	52.64	-1.05
Domestic garden	Woody vegetation	52.65	-1.16
Domestic garden	Woody vegetation	52.62	-1.05
Domestic garden	Woody vegetation	52.62	-1.16
Domestic garden	Woody vegetation	52.63	-1.14
Domestic garden	Woody vegetation	52.65	-1.09
Domestic garden	Woody vegetation	52.62	-1.09
Domestic garden	Woody vegetation	52.62	-1.09
Domestic garden	Woody vegetation	52.65	-1.04
Domestic garden	Woody vegetation	52.62	-1.19
Domestic garden	Woody vegetation	52.60	-1.13
Domestic garden	Woody vegetation	52.64	-1.15
Domestic garden	Woody vegetation	52.68	-1.14
Domestic garden	Woody vegetation	52.60	-1.13
Domestic garden	Woody vegetation	52.64	-1.05
Allotment	Woody vegetation	52.66	-1.11
Allotment	Woody vegetation	52.66	-1.11
Allotment	Woody vegetation	52.63	-1.15
Allotment	Woody vegetation	52.65	-1.13
Allotment	Woody vegetation	52.66	-1.13
Allotment	Woody vegetation	52.66	-1.13
Allotment	Woody vegetation	52.65	-1.13
Allotment	Woody vegetation	52.65	-1.13
Allotment	Woody vegetation	52.60	-1.13
Allotment	Woody vegetation	52.65	-1.06
Allotment	Woody vegetation	52.62	-1.09
Allotment	Woody vegetation	52.62	-1.10
