# Supplementary material for: Urban cultivation in allotments maintains soil qualities adversely affected by conventional agriculture
Source: J Appl Ecol. 2014 Apr 24;51(4):880–9. doi: 10.1111/1365-2664.12254 (PMC4301088; doi:10.1111/1365-2664.12254)

Urban cultivation in allotments maintains soil qualities adversely affected by conventional agriculture

Jill L. Edmondson, Zoe G. Davies, Kevin J. Gaston, Jonathan R. Leake

Appendix S1: Allotment questionnaire

Your Allotment

1. In which site is your allotment situated?

……………….

2. Do you hold more than one plot? If yes, how many plots do you rent?

Yes		No	

3. What is your plot number?

……………….

4. How long have you held your plot?

……………….

5. Which of the following items do you use in your allotment?

Greenhouse heater		Petrol lawn mower		Strimmer	
Petrol chainsaw		Hedge trimmers		Rotavator	
Shredder					

6. Do you compost your allotment material?

Yes		No	

7. Do you add household fruit and vegetable matter to your allotment compost bin?

Yes		No	

8. Have you added any of the types of material listed in the table below to your allotment in the last 5 years? Please estimate amounts of each material added to your allotment each year, based on the equivalent number of filled wheelbarrow.

	0	<1	1-2	3-5	>5		0	<1	1-2	3-5	>5
Commercial compost						Straw					
Your own compost						Topsoil					
Manure						Any other material					
Bark						Please specify the type of other material:					

9. If material is removed from your allotment, is it collected by the Leicester City Council collection scheme?

Yes		No	

10. How frequently do you burn material from your allotment? If never proceed to question 13.

Never		Annually		2–3 times per year		Monthly		Weekly	

11. Can you estimate the volume of material you burn on your plot annually? Please estimate amounts of burnt material, based on the equivalent number of filled wheelbarrows.

<1		1 - 2		3 - 5		6 - 10		>10	
Please estimate the number is you selected >10 category:									

12. Please list the types of material you burn on your allotment?

……………….

13. What types of commercially produced fertiliser do you use on your allotment?

……………….

14. How much of each fertiliser type do you use on your allotment each year?

……………….

15. Have you removed any of the types of material listed in the table below from your allotment in the last 5 years? Please estimate the amounts of each material you remove from your garden each year, based on equivalent number of filled wheelbarrows.

	0	<1	1-2	3-5	6–10	>10		0	<1	1-2	3-5	6–10	>10
Lawn cuttings							Tree prunings						
Hedge/shrub cuttings							Topsoil						
Autumn leaves							Any other material						
Please specify the type of other material:													

16. Could you indicate how many trees over 2m high you have in your allotment?

Deciduous trees are those which lose their leaves in the winter whereas evergreen trees keep their leaves all year round.

Deciduous		Evergreen	

17. Have you had any major tree surgery on your allotment in the last 5 years?

Yes		No	

18. Please specify the number of deciduous and/or evergreen trees removed from your allotment?

Deciduous		Evergreen	

19. What proportion of your allotment is bounded by hedge?

Please use fig. 1 to help you select the most appropriate diagram and indicate your choice in the table below

1		2		3		4		5		6		7		8	

20. Are the hedges in your allotment deciduous or evergreen?

Deciduous		Evergreen	

21. Do you have any hard surfaces in your allotment?

Please use fig. 2 to help you select the most appropriate diagram and indicate your choice in the table below

1		2		3		4		5	

22. When were the hard surfaces in your allotment installed?

....

23. Have you removed any hard surfaces from your allotment?

Please use fig. 2 to help you select the most appropriate diagram and indicate your choice in the table below

1		2		3		4		5	

24. When were the hard surfaces in your allotment removed?

....

25. How many people eat a significant proportion of the produce from your allotment?

....

26. Can you estimate what proportion of the food you eat is produced on your allotment?

Vegetables	0-20%		20-40%		40-60%		60-80%		80-100%	
Fruit	0-20%		20-40%		40-60%		60-80%		80-100%	

Figure 1: What proportion of your allotment is bounded by hedge?

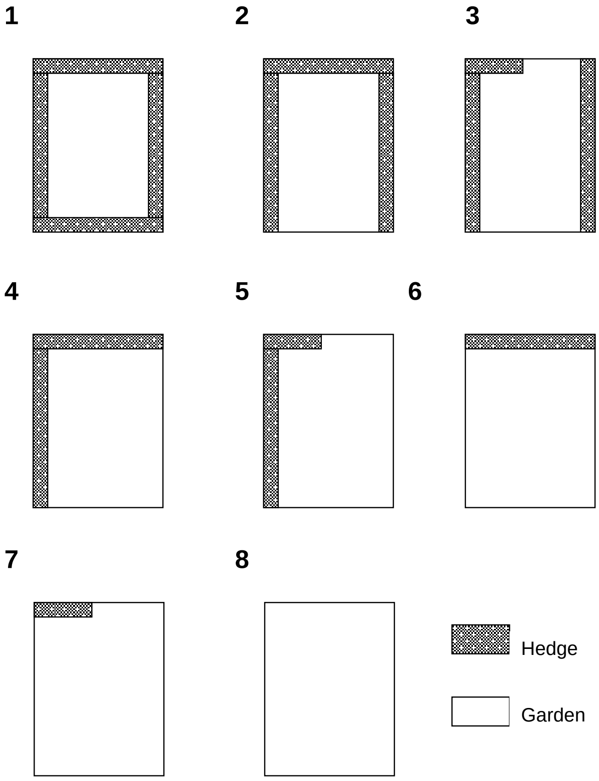

Figure 2: What is/was the proportion of hard surface in your allotment?

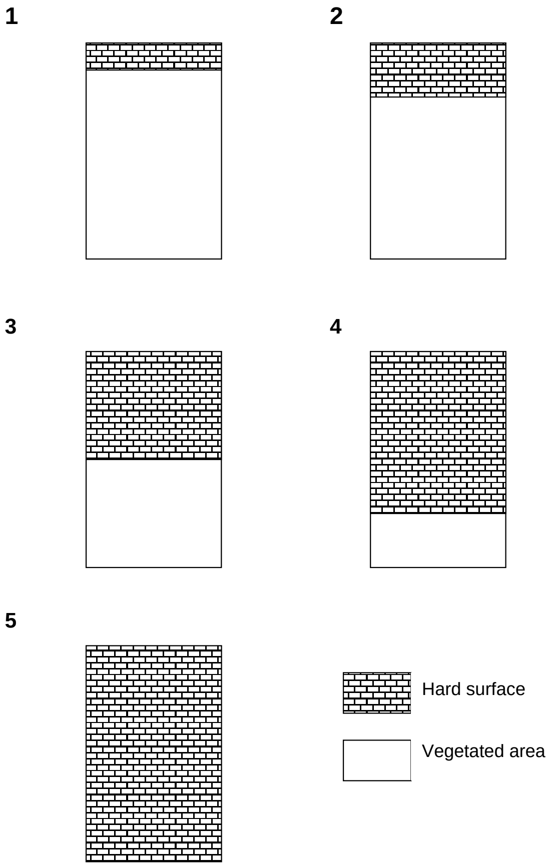

Supplement: Supplementary file 3 — Appendix S1. Allotment questionnaire. [file JPE-51-880-s003.pdf]
